# Comparative perceptions of sexual harassment among athletes across different competitive levels

**DOI:** 10.3389/fspor.2024.1468534

**Published:** 2024-11-28

**Authors:** Sima Zach, Maayan Davidovitz, Rona Cohen

**Affiliations:** ^1^ Levinsky-Wingate Academic College, Wingate Campus, Netanya, Israel; ^2^ School of Education, The Lester and Sally Entin Faculty of Humanities, Tel Aviv University, Tel Aviv, Israel

**Keywords:** sexual harassment, exploitation, coach-athlete relationships, pre-service physical education students, safe sport

## Abstract

**Purpose:**

Despite evidence that sports arenas are grooming ground for sexual harassment, consensus is still lacking what this term constitutes. The aim of this study is to examine how athletes of different levels and non-athletes perceive sexual harassment in sports through the lens of the Institutional Theory.

**Method:**

Hundred and thirty pre-service physical education teachers (competitive and non-competitive athletes) and 53 Olympic athletes, rated 27 items divided into four levels of sexual harassment regarding men coaches' behaviors towards women athletes, on a scale of 1 (does not constitute sexual harassment) to 4 (constitutes sexual harassment to a great extent).

**Findings:**

Non-Olympic athletes rated the behaviors as constituting sexual harassment to a greater degree than Olympic athletes, in all categories, as did women participants compared to man. Both genders rated *sexual harassment and exploitation* as the most severe factor followed by *Sexist behavior*, yet the more competitive the athletes, the less they perceived such behaviors as disturbing. The *Professional contact* factor was rated as the least disturbing, with women rating it as less disturbing than men.

**Conclusions:**

The study underscores the ambiguity surrounding the definition of sexual harassment in sports, emphasizing the necessity for clear boundaries to establish acceptable behavior. Such clarity is essential to ensure that all athletes feel safe within the sporting environment. It emphasizes the importance of zero tolerance for all harassing behaviors, regardless of severity, and the implementation of comprehensive policies and training programs in sport organizations. Moving forward, promoting respect, safety, and awareness, along with ongoing research and evaluation, are crucial for creating inclusive and safe sporting environments.

## Introduction

### Sexual harassment in the sports arena

As a microcosm of society, sports arenas are saturated with incidents of sexual harassment that have a significant impact on athletes in all areas of sports, performance levels, and countries (1–3). The men dominated sports environment encompasses a permissive rape culture and serves as a breeding ground for sexual harassment (4). Some sociological theories even emphasize that the sports arena encourages sexual violence, especially towards younger and older women, yet also towards their man counterparts (5, 6).

Yet ambiguity can be seen in the literature regarding the definition of the term *sexual harassment* and how it is measured. Indeed, universally accepted definitions are lacking for terms such as *sexual harassment*, *abuse*, *exploitation*, *assault*, *harm*, and *violence*, which could be verbal, physical, or emotional. It is also possible for some concepts to overlap or be used synonymously (7). Sexual harassment is considered an illegal act that is perceived as unwarranted, disturbing, threatening, insulting, or offensive (1). It is also generally agreed that sexual harassment involves unwanted sexual attention (8). Brackenridge and Fasting [(9), p.36] define sexual harassment as “unwanted attention on the basis of sex (lewd comments, pinching, touching or caressing, sexual jokes, etc.)” According to Vertommen and colleagues (10), *mild sexual violence*, *moderate sexual violence*, and *severe sexual violence* are three discrete categories. In addition to the intensity of the sexually violent act, scholars also address the frequency of the phenomenon, defining it as *mild* when it occurs only once, or as *severe* when occurring repeatedly (11).

It is well established in the literature that sexual harassment in sports has far-reaching implications, for both athletes, teams, and the integrity of the sports arena in general [e.g., (12–16)]. For example, studies indicate that sexual harassment can lead athletes to being preoccupied with thoughts about the incident (17). The Larry Nassar sexual abuse case involving USA Gymnastics and Michigan State University, for example, has received extensive attention in the literature in recent years [see e.g., (16)]. This case sparked significant public outcry, as it involved an extreme instance in which the team doctor of the United States women's national gymnastics team was accused of hundreds of sexual assaults against woman gymnasts (18). From another perspective, recent findings demonstrate, for example, that among woman students specializing in sports management who experienced sexual harassment, satisfaction with the specialization was lower (19). Women athletes may develop negative attitudes towards men in general, while perceiving their relationship with their coach as ruined. Some athletes may switch to a different field of sports, or even drop out of their elite-level sport or sport activity due to this phenomenon (17). Furthermore, sexual harassment has been found to negatively impact the participation and achievements of younger and older women in sports, in turn harming their quality of life (20). At this point, it is important to emphasize that although most of the literature addressing sexual harassment in sports focuses on abuse perpetrated by men, cases of sexual harassment by women have also been documented (21).

Early studies examined the types of sexual harassment that occur in the sports arena, while attempting to identify the source of harassment. For example, Volkwein and colleagues (22) found that 2% of American women college athletes had been sexually harassed by their coaches, and that nearly one in every five had been subjected to sexist or derogatory remarks. In another study conducted in the UK, one-fourth of the women participants had experienced at least one of the following by their men coaches: demeaning language, verbal intrusion, or physical contact (23). In a study conducted in Belgium, more than one-fifth of the women participants reported that they had experienced at least one of the following behaviors by their coaches: flirting (with them and/or with another team member), making sexual comments about them, and staring (24). As we will discuss later, the lack of gender equality in the sports arena, coupled with the dominant man hegemony in this environment, places women in a particularly vulnerable position regarding their potential experiences of sexual abuse.

### Differences in sexual harassment experiences from the institutional theory perspective

According to the Institutional Theory, which was first introduced in the late 1970s, collective actions are subconsciously impacted by embedded structures, normative social expectations, and cognitive understandings – and to a greater degree than by rational decision making (25–27). One key concept of the Institutional Theory is that both employees and the organization construct a shared understanding of reality through social construction (28). In response to these social constructions, the organization establishes rules and regulations, laws and professional norms, and an ethical code of conduct (27). An organization is characterized by its work patterns, environment, members, and methods – that define how its members should behave in order to control its functioning and production, and as a means for establishing legitimacy and recognition. Specifically, norms develop over time, in an informal and gradual process, during which the members of the organization learn to recognize which behaviors are acceptable and necessary for effectively carrying out their duties (29).

In recent years, observing sports organizations from an institutional perspective has gained momentum, leading to a significant increase in applying the Institutional Theory in sports literature, including studies on sports management (26). Studies have also applied the Institutional Theory as a framework – in an attempt to examine differences between genders within the sports context (30) and address the phenomena of sexual harassment and sexism in the sports arena (3). This concept is particularly relevant in competitive sports, where behaviors such as grooming become institutionalized, as part of the athletes' development process that is perceived as legitimate (31).

Sexual violence does not always arise from issues of desire; it can also occur as an attempt to dominate another party. This is clearer when previous literature emphasizes that coach-athlete relationships should be viewed as relationships of dominance-submission between the parties (32). Coach-athlete relationships, particularly those involving youth athletes, are fertile ground for sexual violence, as a common feature in these arenas is the abuse of power in positions of trust and responsibility (33). Sexual violence can also be perpetuated by unequal power relations between coaches and athletes, or between senior and junior athletes. It is not surprising, then, that in cases of child sexual abuse, the asymmetry in power relations between the parties plays an even more significant role (33).

Not only that, but another aspect that characterizes the sports arena is the increased preoccupation with body aesthetics and the strict nutrition required of athletes to meet performance or social expectations in this environment [see, e.g., (34)]. These elements may also create fertile ground for excessive preoccupation with body care and its externalization, thus serving as an additional catalyst for normalizing an atmosphere of sexual harassment in the sports arena. Norms of this kind, which originate from the practices and cultures within the sports arena, become even clearer when examining the aspects of “cultivation” that many abusers define as part of their medical interventions. This is particularly evident in contexts such as the gymnastics arena in the USA (16), where the use of the “care” framework for some of the care practices was defined by many abusers as part of medical interventions.

It could also be assumed that women athletes may experience sexual harassment differently than men ones. The sports environment is characterized by gender inequality, a predominantly masculine arena in which women athletes are considered distinctly inferior to men ones; in turn, this has created a slippery slope that may have led to the institutionalization of sexual harassment against women athletes. Cunningham and Sagas (30) addresses the inherent nature of gender inequality in sports, arguing that Inequality is an institutionalized element within sport arrangements. In many cases, this inequality is identified with the man hegemony that dominates the sports arena [e.g., (35)]. There is therefore fertile ground for sexual harassment in this arena, especially as man dominated organizations and professions have a high prevalence of sexual harassment and sexism incidents (3). As a result, it is not surprising that women experience more sexual harassment than men (10). Earlier findings have also demonstrated this empirically within the Israeli context (36).

In the context of sports, the term *grooming* emphasizes a strategy whereby coaches strive to build their athletes’ trust in them, as a means for consciously persuading them to engage in sexual acts (9, 33, 37). A study conducted by Bisgaard and Støckel (38), using the athletes' own words (i.e., realistic narrative), offers an in-depth understanding of sexual harassment and abuse in sports.

These narratives indicate how such grooming behaviors are often incorporated into the daily culture and practices of sports. The particularly close athlete-coach relationship that exists in competitive sports institutionalizes the phenomena of *nurturing*, and in turn, enables the legitimization of the sexual harassment phenomenon in this arena. In this context, while *sexual harassment* is a non-normative phenomenon, the abuse itself may be perceived by the victim as acceptable (9). The scoping review conducted by Gaedicke and colleagues (33) illustrates – inter alia – how certain aspects of the grooming process within the coach-athlete context contribute to this phenomenon. For example, they point out how building a friendship between coaches and athletes may blur the lines between legitimate behavior on both sides. They also found that the grooming process “normalizes” behaviors of sexual harassment and sexual exploitation – especially through the coach-athlete trust and interdependence that develop.

As competitive and high-achieving sports have been found to provide fertile ground for grooming relationships between coaches and athletes (37), this phenomenon seems to have become institutionalized in the sports arena, and its prevalence is likely to continue to exist. It has long been recognized that the broader context in which sports operate is critical to understanding how norms and culture shape practices within the sports arena. The coach-athlete literature, particularly the “darker side” work of Bartholomew and colleagues (39), focuses on need-thwarting practices in this environment. This underscores the importance of understanding the context in which athletes operate to comprehend grooming phenomena as a foundation for experiences of sexual harassment in the sporting environment. Owton and Sparkes (37), for example, empirically demonstrate how an athlete who was groomed by her coach experienced sexual abuse in the continuation of their relationship. Through their autoethnographic research focusing on this woman athlete, they illustrate how structural conditions and power relations in the sports arena create an “enabling” context for the phenomena of sexual abuse and exploitation. However, due to current efforts to educate and raise awareness through social media and women speaking out, it is likely that these efforts will help reduce the phenomenon of sexual harassment in sports. Recent studies reveal that the “MeToo” campaign has far-reaching consequences for exposing this issue and narrowing its prevalence in the sports arena (40, 41) similar to its impact in other social arenas. Additionally, the voices of athletes regarding cases of sexual harassment in sports contribute to changing the scope of the phenomenon. A prominent example can be identified in the reports of U.S. Gymnastics athletes in recent years. When athletes speak out about issues of sexual abuse in sports, media attention increases, and the cases gain significant public resonance (16). However, previous studies emphasize that the manner of media coverage is also crucial in avoiding the over-reporting of information about these abuse cases, which can inadvertently victimize the athletes through a victimization lens (16). No less importantly, previous literature emphasizes the institutional deficiencies that allow, or at least do not fully prevent, the occurrence of this phenomenon and highlights the importance of the institutional regulations required by decision-makers to minimize it (42). There is a clear consensus that it is the role of policy makers to ensure the necessary legislation and implementation to optimally address the issue of sexual harassment in sports (42).

In high-achieving sports, coaches and athletes have particularly close relationships, with the former having vast control and influence over the latter. While such power enables successful athletic performance, it also provides fertile ground for sexual harassment and exploitation (43–45). As such, athletes of varying levels of participation could perceive sexual harassment differently. Furthermore, the literature highlights a culture of silencing and nurturing that encourages athletes to accept and even defend sexual abuse as normal occurrences (3), resulting in the under-reporting of such incidents in competitive sports. In this context, it is important to emphasize the power structure of the sports institution, where the majority of women athletes are accompanied by men coaches, and the majority of man athletes are accompanied by men coaches as well. The institutionalized heterosexual norm creates power dynamics that place women athletes at a significantly higher risk of sexual harassment and abuse than man athletes.

Based on this literature review, the aim of this study is to understand how pre-service physical education (PE) teachers, coaches, and athletes of different levels perceive sexual harassment of men coaches toward women athletes, with an emphasis on differences in perceptions between men and women – examined in light of the Institutional Theory. We assume that women athletes will be affected by sexual harassment differently than man athletes. As such, we present the following two research hypotheses:

H1: Pre-service PE teachers who participate in competitive sports will perceive sexual harassment more severely than those who are Olympic athletes and those who do not participate in competitive sports.
H2: Women athletes and non-athletes will express a different interpretation than men athletes/non-athletes on whether or not specific behaviours from men coaches are sexual harassment.

This study addresses a critical gap in our understanding of how sexual harassment is perceived within the sporting community. By examining the perceptions of pre-service physical education teachers, coaches, and athletes at different competitive levels, this research provides a comprehensive analysis of how sexual harassment is understood and interpreted across the spectrum of sports involvement. The inclusion of both men and women participants allows for a nuanced exploration of gender differences in these perceptions, offering valuable insights into how experiences and socialization within sports contexts may influence attitudes towards sexual harassment.

This research is particularly significant in its application of Institutional Theory to examine how organizational structures and norms within the sports world may shape perceptions of sexual harassment. By comparing the views of non-competitive physical education students, those engaged in competitive sports, and elite Olympic athletes, the study illuminates how different levels of immersion in sporting institutions may affect one's understanding and recognition of sexual harassment. This multi-level approach not only contributes novel findings to the existing literature but also has important implications for policy development and educational initiatives aimed at preventing sexual harassment in sports. The focus on pre-service physical education teachers and coaches is especially valuable, as these individuals will play pivotal roles in shaping future sporting environments and fostering safe, inclusive spaces for athletes of all levels.

## Methodology

### Participants

The study included 183 participants (119, 65% women, 64, 65% men), aged 18–49 (*M* = 26.46; SD ± 5.72), comprised of 53 Olympic athletes, and 130 pre-service PE teachers from a college of education in Israel who were categorized as either competitive athletes (*n* = 83) or non-competitive athletes/non-athletes (*n* = 47).

Olympic athletes are those who compete at the highest level in their sport, striving for qualification and success in the Olympic Games, which requires rigorous training and dedication. Non-Olympic athletes, on the other hand, may participate in various sports at different levels without the specific goal of competing in the Olympics, often focusing on recreational, amateur, or local competitions. In the group of *Olympic athletes*, 60.6% participated in martial arts, 12.1% in swimming, 6.1% in team ball games, and 3% in track and field; the remaining 18.2% participated in other fields of sports. On average, they had begun engaging in sports at the age of 7.25 (SD ± 3.57) and had retired from competitive sports at the average age of 26.14 (SD ± 5.88). At the time of the study, 34% had been competitive athletes in the past and were currently working as coaches – with 50.9% of them working as coaches in the fields of artistic gymnastics, tennis, judo, basketball, swimming, weightlifting, cycling, Olympic shooting, gymnastics, water polo, rock climbing, and triathlons.

Competitive athletes are primarily driven by the desire to win and improve performance, often engaging in structured training regimens and participating in organized events, whereas, non-competitive athletes participate in sports for enjoyment, fitness, and social interaction, emphasizing personal fulfillment over competitive outcomes. In the group of *competitive athletes*, 75% participated in team ball games, 7.1% in martial arts, 7.1% in track and field, and 3.6% in swimming; the remaining 7.1% participated in other fields of sports. On average, they had begun engaging in sports aged 10.7 (SD ± 4.53). At the time of the study, 39.3% were working as competitive sports coaches.

Non-Olympic athletes engage in sports or physical activities for enjoyment, fitness, or competition at various levels but do not aim for Olympic qualification, whereas non-athletes, do not participate in sports regularly and may prioritize other interests, often leading a more sedentary lifestyle. In this group of *non-competitive athletes* (or non-athletes), 53.9% reported that they had been involved in competitive sports in the past. On average, they had begun engaging in sports at the average age of 9.58 (SD ± 4.37) and had retired from competitive sports at the average age of 17.67 (SD ± 3.1). At the time of the study, 64.7% of this group were working as non-competitive sports coaches. The pre-service PE teachers who were non-athletes was relatively bigger than the other two groups in the study. These future teachers will hopefully be able to instill relevant values in their school students. Therefore, clarifying their perceptions is imperative with a follow up educational program.

### Questionnaire

First, the participants were asked a number of general background questions, such as age and gender, followed by questions regarding their athletic experience, including type of sport, athletic level of performance, and years of experience. Next the participants were asked questions about what they consider to be *sexual harassment.* The original questionnaire was compiled by Volkwein et al. (22) in a study on 200 student-athletes at three colleges in the USA. The questionnaire was later translated into Hebrew in Israel and validated in a study on pre-service PE teachers (46). The questionnaire includes 27 items regarding a man coach's behavior towards a woman athlete. For each item, the participants were asked to rate the extent to which they perceive this behavior as constituting sexual harassment, on a scale of 1 (not at all) to 4 (to a great extent), or alternatively 5 (don't know) – a rating that was not included in the statistical analyses. Using the Varimax method, Fejgin and Hanegby (46) conducted factor analysis, resulting in the following four dimensions:

1. Factor 1. *Severe harassment and exploitation* (10 items) 0.873.
2. Factor 2. *Between concern and interest* (6 items) 0.854.
3. Factor 3. *Sexist behavior* (5 items) 0.834.
4. Factor 4. *Professional contact* (6 items) 0.790.

The first factor, *Severe harassment and exploitation*, relates to physical expressions (such as pinching the athlete's butt or kissing her on the mouth) and verbal behaviors (such as sexual suggestions or expressing an interest in the athlete's sex life.) In this study, the internal reliability of this factor was found to be Cronbach's *α* = 0.83. The second factor, *Between concern and interest*, relates to disturbing behaviors regarding the athlete's professional or private life (such as the coach inviting her to his home for coffee or expressing an interest in her plans for the weekend.) In this study, the internal reliability of this factor was Cronbach's *α* = 0.85. The third factor, *Sexist behavior*, relates to inappropriate verbal behavior (such as complimenting the athlete on her appearance or telling rude jokes.) In this study, the internal reliability of this factor was Cronbach's *α* = 0.84. The fourth and final factor, *Professional contact*, relates to certain aspects of the coach's training (such as physical contact while demonstrating or teaching or when expressing joy following the athlete's victory.) In this study, the internal reliability of this factor was Cronbach's *α* = 0.80.

### Procedure

The pre-service PE teachers were contacted via the mailing lists of the authors' affiliated academic institution; the Olympic athletes were contacted via the Olympic Committee of Israel. The study was approved by the Institutional Review Board at the authors' affiliated academic institution (No. 257, 2020). After submitting a signed written consent form, the participants received the study questionnaire via email. Anonymity was ensured throughout the study.

### Data analysis

First, we present descriptive statistics. Then, to test main effects and combined effects of gender and athletic level of performance, two-way analysis of variance (ANOVA) was conducted for each of the four factors addressed in the questionnaire.

## Results

First, mean scores (M) and SD were calculated for the rating of each of the 27 man coach–woman athlete behaviors, on a scale of 1–4. For each item, we also examined the percentage of man participants and of women participants who rated an item as 4, indicating that the specific man coach–woman athlete behavior constitutes sexual harassment to an extreme degree (Table 1).

**Table 1:** Participants’ perceptions of the factors and items as constituting sexual harassment (*N* = 183).

			Olympic Athletes *n*-53		Pre-service PA teacher athletes *n* = 83		Pre-service PA teacher non-athletes *n* = 47	
			Rated the item as 4 (to a great extent)					
	*M*	SD	Ma% *n* = 14	Fe% *n* = 39	Ma% *n* = 42	Fe% *n* = 41	Ma% *n* = 8	Fe% *n* = 39
Severe harassment and exploitation	**3** **.** **75**	**0**.**23**						
1. Shows sexual interest in the athlete	3.85	0.44	84.62	94.59	80.49	85.44	75.00	89.74
2. Proposes sexual encounters without reward for agreement/threat for rejection	3.83	0.49	83.33	89.19	85.37	86.67	87.50	89.74
3. Tells the athlete about his sex life	3.78	0.52	71.43	86.49	78.05	80.24	75.00	89.74
4. Stares at the athlete's breasts	3.79	0.46	64.39	81.08	82.93	82.93	62.50	87.18
5. Gives the athlete a back/shoulder massage for fun	3.23	0.88	28.67	22.86	55.26	60.34	50.00	52.63
6. Pinches the athlete on her behind	3.9	0.34	85.71	91.89	92.68	90.24	75.00	97.37
7. Asks the athlete about her sex life	3.83	0.4	57.14	89.19	80.00	81.55	75.00	97.30
8. Proposes sexual encounters, with reward for agreement/threat for rejection	3.93	0.25	92.91	94.59	92.68	91.56	100.00	94.74
9. Kisses the athlete on the mouth	3.92	0.29	92.91	94.59	92.68	91.56	75.00	97.30
10. Caresses the athlete	3.5	0.71	35.71	48.65	65.00	61.73	62.50	73.00
Between being concern and showing an interest	**2**.**41**	**0**.**36**						
11. Invites the athlete to train at his home	2.14	1.10	7.14	3.03	21.62	19.73	25.00	28.57
12. Invites the athlete to his home for coffee	2.18	1.01	7.70	5.41	7.50	11.34	0.00	33.33
13. Asks the athlete what she does in her spare time	2.08	0.94	0.00	2.78	12.82	8.87	0.00	15.79
14. Invites the athlete out for dinner	3.04	1.07	21.43	25.71	46.34	50.00	50.00	68.42
15. Asks the athlete about her plans for the weekend	2.55	1.09	14.30	13.51	31.71	28.06	37.50	30.77
16. Invites the athlete to a nearby café for lunch	2.44	1.00	7.14	5.56	20.00	22.54	25.00	18.42
Sexist behavior	**2**.**57**	**0**.**42**						
17. Talks about what he likes to do in his spare time	2.02	0.93	0.00	2.78	5.00	8.97	12.50	15.79
18. Calls the athlete by a pet name (such as “sweetie” or “honey”)	2.6	0.96	14.3	19.44	17.07	17.07	37.50	28.95
19. Tells the athlete about his plans for the weekend	2.27	0.99	0.00	5.71	19.51	17.07	37.50	15.79
20. Compliments the athlete on her looks	2.96	0.82	21.43	5.56	30.00	32.13	25.00	44.74
21. Makes derogatory remarks about women	2.98	0.90	28.61	27.78	29.27	24.74	37.50	51.35
Professional physical contact	**2**.**31**	**0**.**84**						
22. Touches the athlete on her shoulder or arm while giving explanations	1.69	0.88	0.00	2.78	5.35	6.35	0.00	13.21
23. Sits or stands close to the athlete when talking in the office	2.21	0.97	0.00	5.56	7.32	11.04	12.50	20.51
24. Closes the door when talking in the office	2.33	1.02	0.00	2.78	20.00	18.85	25.00	23.68
25. Places his hand on the athlete's shoulder or arm when greeting her	2.17	1.00	14.30	2.78	14.67	14.87	37.50	15.4
26. Kisses the athlete on her cheek	3.92	0.29	57.14	22.22	58.5	67.16	62.50	64.1
27. Hugs the athlete after winning a competition	1.56	0.85	0.00	0.00	5.00	5.00	12.50	10.5

*M* = mean scores; SD = standard deviation; Ma = Males; Fe = Females.% Rated the item as 4 (to a great extent).

Bold numbers represent Means and SDs of the questionnaire' factors.

The factor that was most perceived as constituting sexual harassment was *Severe harassment and exploitation*, with the highest mean score (M = 3.75) and the smallest SD (SD = 0.23), thereby indicating a high level of agreement among the participants regarding the seriousness of the items that were presented in this factor. In general, women participants tended to perceive these behaviors as more disturbing than man ones. This difference between genders was especially prominent in the group of non-competitive athletes and was much smaller in the group of Olympic athletes. The most noticeable differences between the three groups of participants were in relation to item 5, i.e., *The coach gives* *the athlete a back or shoulder massage just for fun*, whereby 60% of the competitive athletes and 52% of the non-competitive athletes rated this item as 4 (i.e., extreme sexual harassment), while only 28% of the Olympic athletes did so. Considerable differences were also seen regarding item 10, *Caresses the athlete*, whereby 73% of the non-competitive athletes and 62% of the competitive athletes rated this behavior as extremely disturbing (4 on the rating scale), compared to only 35% of the Olympic athletes.

The next factor that was most perceived as constituting sexual harassment was *Sexist behavior*, with an average score of 2.57 (SD ± 0.42). In the group of Olympic athletes, no differences were seen between genders in their scores, except for item 20, *Complimenting the athlete on her appearance*, which was perceived by man participants as more disturbing than by woman ones; however, in the group of non-competitive athletes, women participants perceived this item as more disturbing than their man counterparts. Moreover, participants from the non-competitive group perceived the behaviors in this factor as very disturbing, while those from the Olympic group perceived them as least disturbing. The largest gap between the three groups was seen in item 20, *Complimenting the athlete on her appearance*, with 44% of the participants in the non-competitive group rating this as 4 (constitutes sexual harassment to a great extent), compared to 32% in the competitive group, and only 5% in the Olympic group.

The mean score for the *Between concern and interest* factor was *M* = 2.41 (SD ± 0.36), indicating that the behaviors presented in this factor are perceived as less disturbing than those presented in the previous two. In this factor, both genders rated these behaviors fairly similarly. However, for four statements, man participants perceived these behaviors as more disturbing than woman ones, while for three behaviors, women participants in the group of non-competitive athletes perceived these as more harassing than their man counterparts. In general, participants from the non-competitive group rated the behaviors in this factor as most disturbing while those from the Olympic athletes group rated them as least disturbing. The largest difference between participants was seen in statement number 14 *Invites the athlete out for dinner*, with 68% of participants from the non-competitive group rating this as extremely disturbing (score 4), followed by 50% of participants from the competitive group, and finally, only 25% of the Olympic athletes perceived this behavior as disturbing.

The factor that was least perceived as constituting sexual harassment in the sports arena was *Professional contact* (*M* = 2.31; SD ± 0.84). Interestingly, four of the six items in this factor were not rated as 4 (to a great extent) by any of the man participants in the Olympic group. In fact, some of these six items were rated as 1 (not at all disturbing), including item 27, *hugs the athlete after winning a competition*, and item 22, *touches the athlete on her shoulder or arm while giving explanations.* On the other hand, a high rate of participants perceived item 26, *Kisses the athlete on her cheek*, as highly disturbing. In the group of Olympic athletes, the following two items were perceived by men participants as more disturbing than by women participants: item 25, *Places his hand on the athlete's shoulder or arm when greeting her*, and item 26, *Kisses the athlete on her cheek*. In the two non-Olympic groups, differences between genders were relatively small; moreover, 32% of the Olympic athletes perceived this factor as not disturbing, except for the above-mentioned item 26.

### Gender and sports

**Figure 1:**
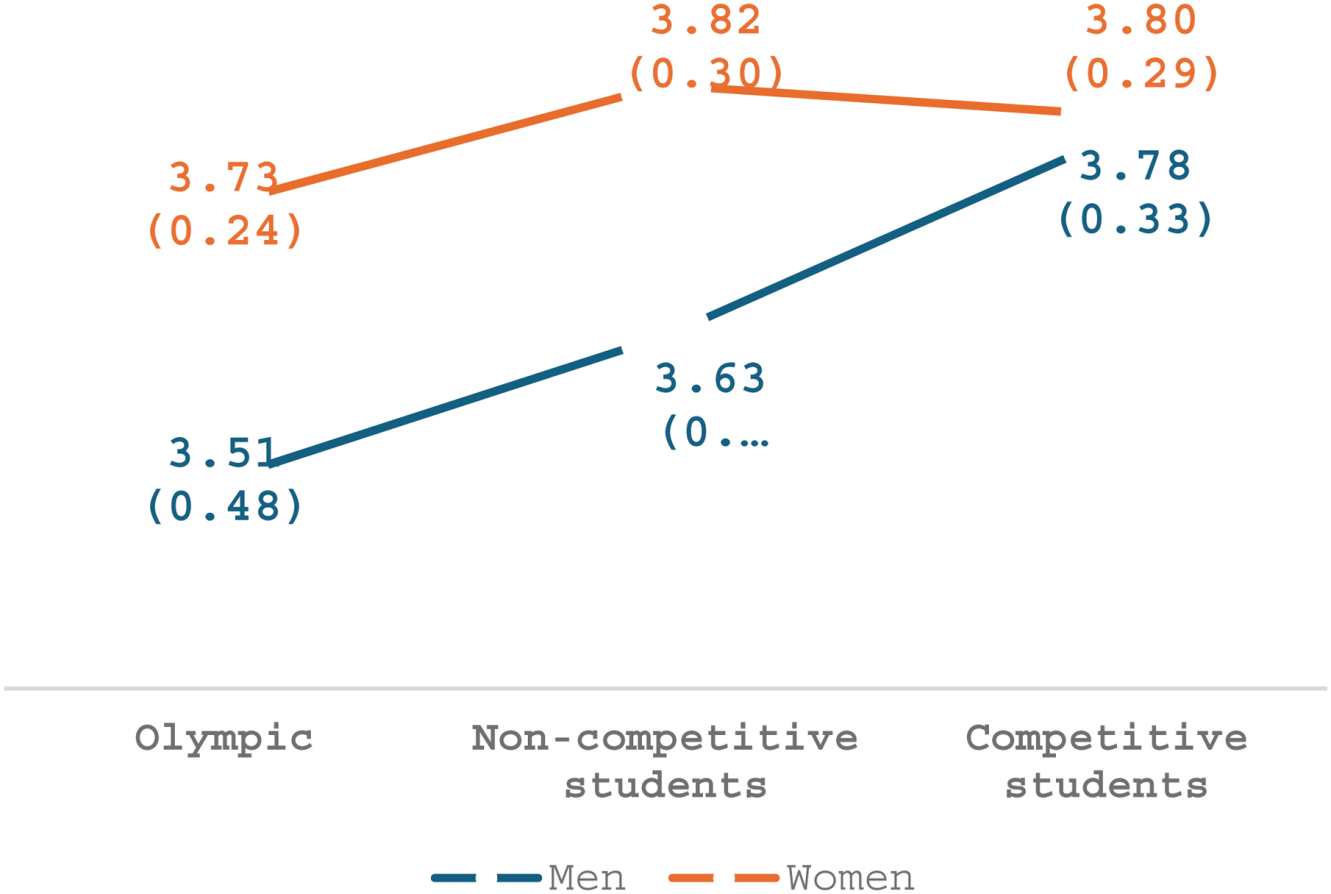
Severe harassment and exploitation.

To test main effects and combined effects of gender and athletic level of performance, two-way analysis of variance (ANOVA) was conducted for each of the four factors addressed in the questionnaire. Regarding the factor, *Severe harassment and exploitation*, a main effect was seen for participants' athletic level of performance [*F*_(2,174)_ = 3.67, *p* = .03, *η*2 = .04], with pairwise comparison analysis showing that participants in the competitive group perceived these items as constituting sexual harassment to a greater extent than those in the Olympic group. However, no differences were found between the non-competitive group and the Olympic group. As seen in Figure 1, a main effect was also found for gender in this factor [*F*_(1,174)_ = 6.02, *p* = .01, *η*2 = .03], whereby women participants perceived these behaviors as more disturbing than man ones. No interaction was seen between gender and athletic level (*p* = .21).

**Figure 2:**
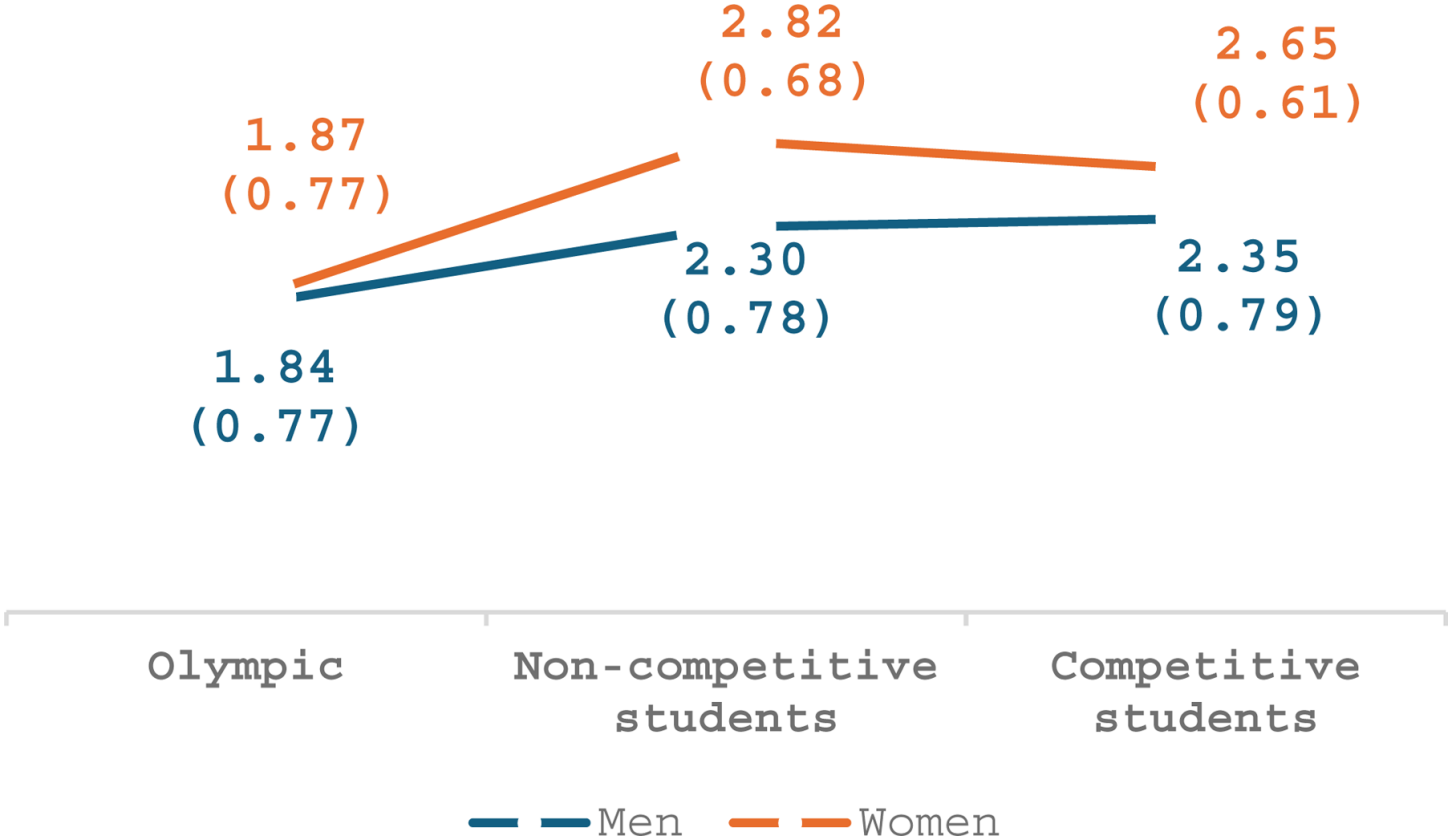
Between being concern and showing interest.

Regarding the second factor, *Between concern and interest*, a main effect was seen for participants' athletic level of performance [*F*_(2,174)_ = 12.44, *p* < .001, *η*2 = .12], whereby both groups of non-Olympic participants perceived these behaviors as more disturbing than the Olympic athletes. As seen in Figure 2, a main effect was also found for gender [*F*_(1,174)_ = 4.53, *p* = .03, *η*2 = .02], whereby women participants perceived these behaviors as more disturbing than man ones. No interaction was seen between gender and athletic level (*p* = .39).

**Figure 3:**
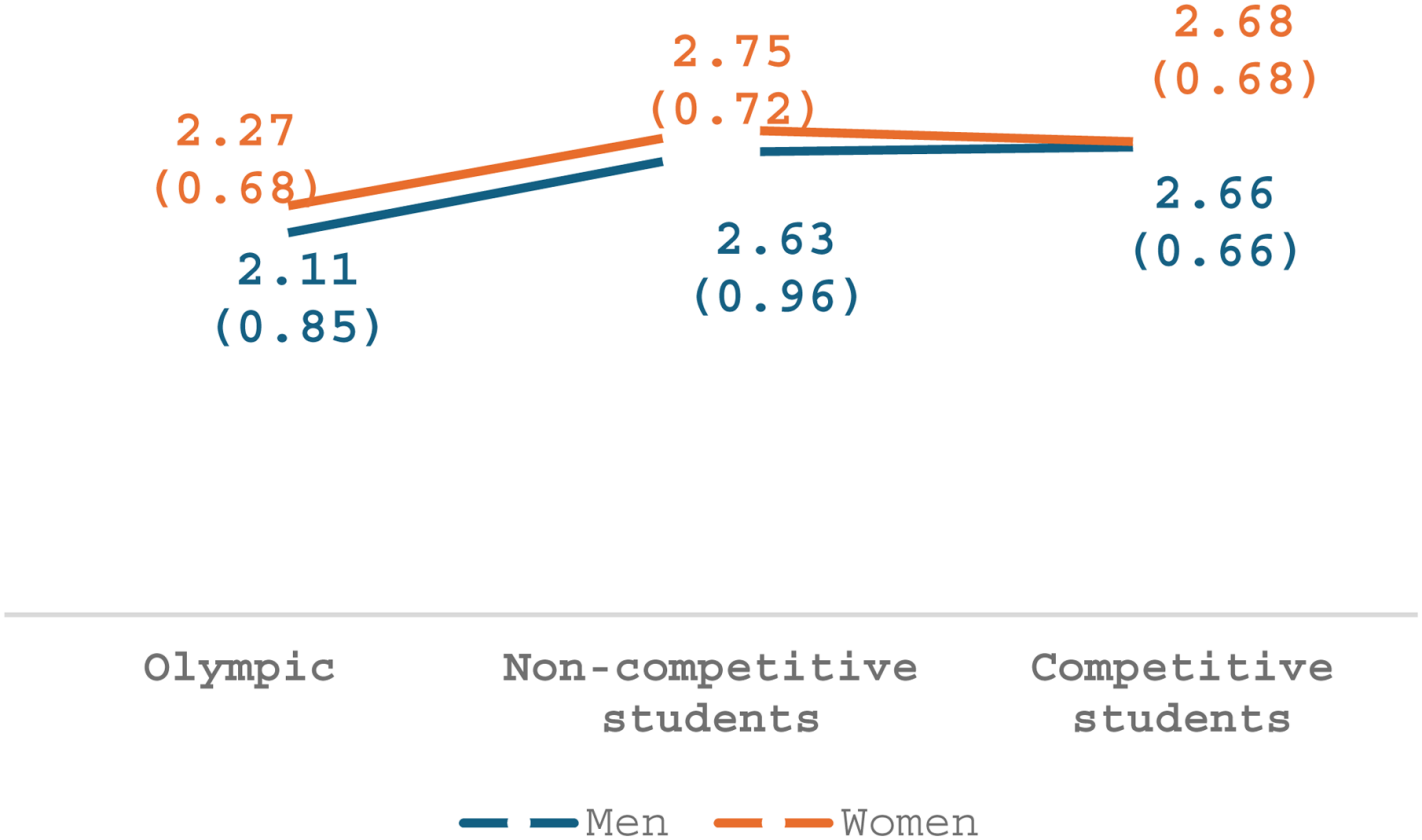
Sexist behavior.

Regarding the third factor, *Sexist behavior*, a main effect was seen for participants' athletic level of performance [*F*_(2,173)_ = 6.69, *p* = .002, *η*2 = .07], whereby both non-Olympic groups perceived these behaviors as more disturbing than Olympian athletes. As seen in Figure 3, no main effect was seen for gender (*p* = .43) and no interaction was seen between gender and athletic level (*p* = .86).

**Figure 4:**
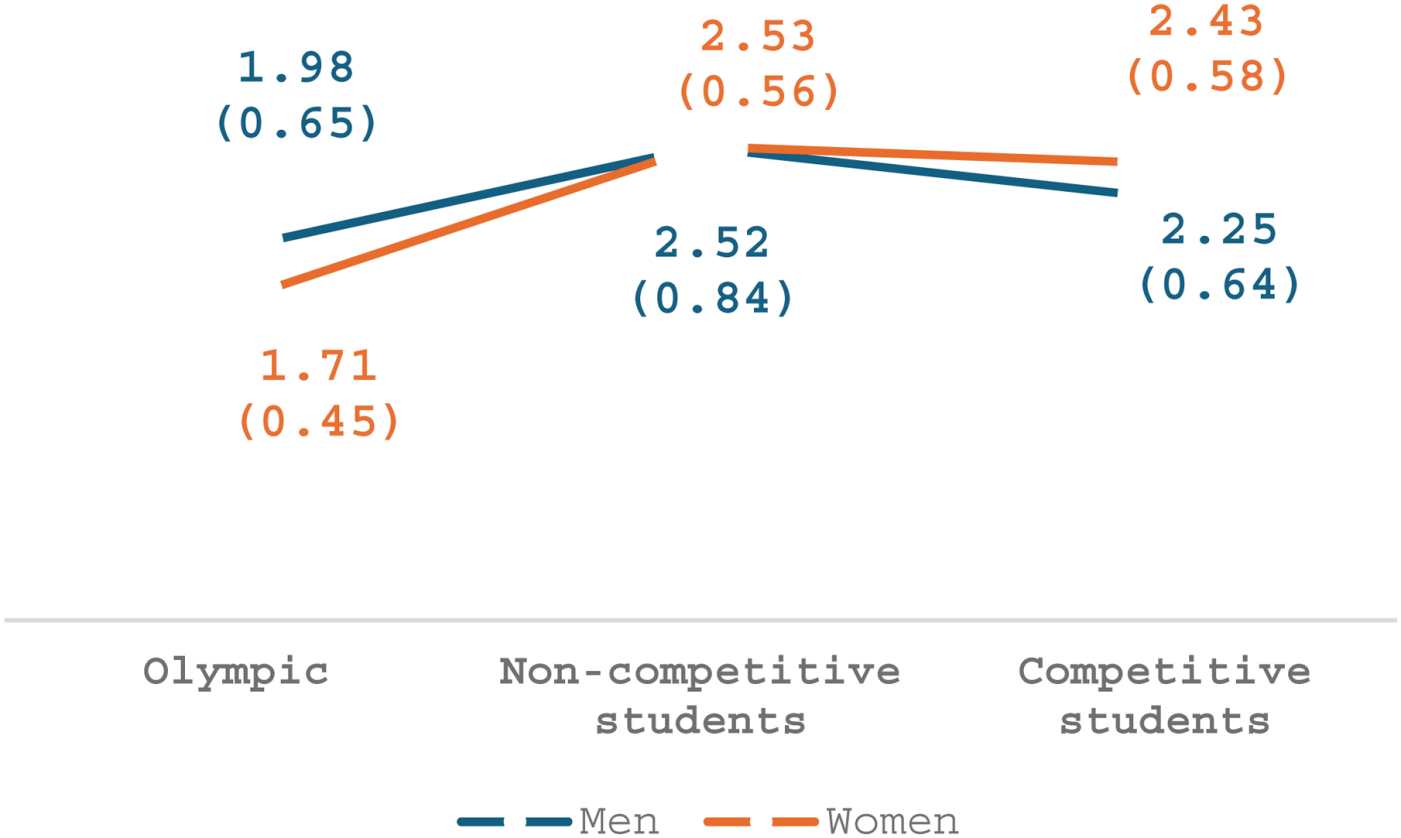
Professional physical contact.

Regarding the fourth and final factor, *Professional contact*, a main effect was seen for participants' athletic level of performance [*F*_(2,174)_ = 13.47, *p* < .001, *η*2 = .13], whereby both groups of non-Olympic participants perceived these behaviors as more disturbing than Olympic athletes. As seen in Figure 4, no main effect was seen for gender (*p* = .79) and no interaction was seen between gender and athletic level of performance (*p* = .13).

## Discussion

This study examined how Olympic athletes and pre-service PE teachers perceive behaviors of men coaches towards women athletes, within the broad umbrella of the term *sexual harassment*. Such analysis is especially important in light of the lack of consensus regarding what constitutes sexual harassment in general and in the field of sports in particular. In this research, an emphasis was placed on differences in perceptions between competitive athletes, non-competitive athletes, and Olympic athletes, and on differences between genders within and between these groups. The results of the current study reinforce earlier findings regarding differentiated attitudes towards sexual harassment [e.g., (36, 47)]. For example, the *Severe harassment and exploitation* factor and the *Sexist behavior* were rated as more disturbing than the *Between concern and interest* factor and the *Professional contact* one. This indicates that participants do not perceive all behaviors as constituting sexual harassment to the same degree. Brackenridge (47) explains that the terms *sexual exploitation*, *sexual harassment*, *sexual violence*, and *sexual assault* are often perceived as a continuum, which could explain why not all behaviors elicit the same response. Yet while all groups of participants and both genders perceived items in the *Severe harassment and exploitation* factor as the most disturbing, followed by those in the *Sexist behavior* factor, differences were seen between men and women, and between the three groups of participants according to their level of sports performance.

Indeed, our findings highlight the lack of consensus regarding what is considered sexual harassment and/or its severity. This could be explained in part using the Institutional Theory, whereby the sport as an organization provides a set of rules that define how its members should behave, as a means of control and for establishing basic legitimacy and recognition (29). Moreover, competitive sports tend to institutionalize behaviors such as grooming, as part of a legitimate development process for athletes (38), which could explain why certain behaviors were perceived by the Olympic athletes as legitimate, yet were perceived as disturbing by the pre-service PE teachers who are not part of a sport institutions, but rather are part of a college institution that emphasizes different values (48).

Interestingly, the behaviors that are described in the *Between concern and interest* factor were rated as less disturbing than those in the *Severe harassment and exploitation* one. Moreover, both man and women participants perceived these behaviors at a fairly similar rate. However, the Olympic athletes perceived the behaviors in the *between concern and interest* factor as least disturbing, while the non-competitive participants perceived them as most disturbing. The name of the factor points at a lack of distinction, indicating a type of continuum. Hassall et al. (49) claims that some coach-athlete behaviors can be perceived as ambiguous, meaning that they may or may not be construed as sexual harassment. It is evident from the current findings that athletes from different levels of sports do not share the same attitudes towards different coach-athlete behaviors, perhaps reflecting this ambiguity. For example, the item, *Invites* *the* *athlete out for dinner* was perceived as three times more severe and disturbing by the non-competitive group than by Olympic athletes – two populations that do not share the same organizational culture. However, when it is not clear what is an accepted or unaccepted behavior, and the line between support/closeness/contact becomes blurred, interpretations may vary. Carstensen (50) therefore claims that sexual harassment should not be defined solely by the victim's interpretation, but should also be addressed in relation to the grey area, the context of the behavior, and the continuum of behaviors. The researcher also emphasizes the need to concentrate on structural dimensions as a means for increasing gender equality in organizations and other work domains.

As stated, the *Professional contact* factor was perceived as the least disturbing, and women participants perceived the behaviors in this factor as *less* disturbing than man ones, especially within the group of Olympic athletes. As such, it seems that women may not perceive intimate body gestures, such as hugs and kisses on the cheek, as disturbing, whereas men do. These findings have several possible explanations. First, as suggested by Taylor et al. (3), the culture of silencing and nurturing that exists in sports organizations encourages athletes to accept and even defend sexual abuse as a normal occurrence. As such, such behaviors are less likely to be reported and/or to be perceived as disturbing by athletes from competitive sports organizations. Moreover, different participants from different backgrounds may perceive certain behaviors to be in the grey zone along the continuum of harassment or find ambiguity in relation to what is considered harassment.

It is possible that differences exist between men and women regarding the perception of what constitutes a “red line.” That is, when the issue of sexual harassment was first raised as a widespread phenomenon in all its layers, it was initially directed towards men harassing women. The process of conceptual change and the internalization of its implications took years, and once it was internalized, it became apparent that men, at least at a perceptual level, are aware of the problematic nature of this phenomenon. In contrast, women, who were traditionally on the receiving end of such harm, find themselves in a position where their ability to discern when and how they are threatened includes a certain level of tolerance. Moreover, it is important to remember that there are also cultural differences regarding what is perceived as intimate proximity and which statements are considered compliments or harassment. Lastly, there is the expression, “Once burned, twice shy,” meaning that when striving for change, the full range of behaviours is addressed.

This study offers important insights regarding how people from different levels of athletic performance, and how people from the different genders, perceive sexual harassment. Yet despite its contribution, some research limitations should be addressed. First, both the pre-service PE teachers and the Olympic athletes who participated in the study volunteered to do so, and as such, may be more aware of the research subject matter than those who chose not to take part in the study. In addition, the questions were regarding the behavior of men coaches toward women athletes whereas both genders could be included in all questions. Therefore, generalization of the results should be performed with caution. It should be noted that for both women and men athletes, some research suggests that those with men coaches are more tolerant of sexually harassing behaviours while those with women coaches are less tolerant of these same behaviours (51). Also, the research participants represent a narrow segment of the athlete population, particularly absent are those from high stakes, hypermasculinized and physical sports for the most part.

Following the findings of the current study, whereby less severe behaviors may be excused, while more extreme behaviors may be condemned, we join Carstensen' (50) suggestions regarding the importance of eliminating ambiguity and grey areas in order to increase the important aim of achieving gender equity in sport organizations (and in other workplaces.) Therefore, all harassing behaviors should be treated in the same manner, i.e., with zero tolerance (although punishment for such behaviors can vary.) Still, we should keep in mind that although some behaviors are consensual and mutually fulfilling, if “no” means no, does “yes” mean yes? (52). Indeed, such behaviors must be addressed and dealt with in the literature, rather than remaining within the grey area.

Despite the expectation that with current efforts to education and heighten awareness through social media and women speaking out, sexual hassment have become institutionalized in the sports arena, and its prevalence is likely to continue to increase (53, 54). One possible explanation for the increase in sexual harassment in sports is the pervasive culture that has long been entrenched in many athletic organizations. Despite efforts to promote awareness and provide education, these initiatives often face resistance due to deeply ingrained attitudes and beliefs. Moreover, the lack of a universally accepted definition of sexual harassment complicates efforts to address and combat it. This ambiguity can lead to inconsistent reporting and enforcement, making it difficult to hold perpetrators accountable (53, 55). Additionally, while social media and public discourse have empowered more victims to come forward, they have also highlighted the extensive nature of the problem, revealing cases that might have previously gone unnoticed. This increased visibility may give the impression that sexual harassment is on the rise, even as awareness and intolerance of such behavior grow. Furthermore, the efficacy of regulations and their enforcement is fundamental to creating a safer environment in sports. Without stringent policies and consistent enforcement, attempts to curb sexual harassment may fall short. Effective regulations need to be clearly defined, universally applied, and backed by serious consequences for violations to foster a genuine shift in the sports culture. In summary, while there are ongoing efforts to address sexual harassment in sports through education and awareness, the issue remains prevalent due to cultural entrenchment, ambiguous definitions, and inconsistent enforcement of regulations. Addressing these underlying challenges is essential for any substantive progress in reducing sexual harassment in the sports arena.

Disagreement over the definition of sexual harassment creates a context where different interpretations are applied to behaviours by individuals in different positions. Such a context prevents the establishment of rules and regulations that would apply to everyone in order to reduce or prevent such behaviours. Furthermore, in terms of punishment, if there is no consensus regarding the severity of the actions, it becomes impossible to reach agreement on appropriate penalties, resulting in behaviours for which there are no deterrents.

The conclusions drawn from the current study highlight the lack of consensus regarding what should be defined as sexual harassment in sports. Despite similar perceptions among man and women participants, context was found to play a significant role in how sexual harassment is perceived and interpreted. The findings suggest that behaviors considered less severe may be excused, while more extreme behaviors may be condemned. This ambiguity in defining and addressing sexual harassment in sports can hinder efforts to achieve gender equity within sport organizations and other workplaces.

From conducting this research, it is evident that there is a need to eliminate ambiguity and grey areas in defining and addressing sexual harassment in sports. All harassing behaviors should be treated with zero tolerance, although the appropriate punishment may vary. It is crucial to address consensual behaviors that may still be harmful and ensure that boundaries are respected. This research can inform sport organizations and educational programming by emphasizing the importance of clear policies and training on sexual harassment prevention and response.

It is important to note that this study exclusively focuses on the experiences of adults, and that the experience of harassment may manifest differently and have distinct effects on child athletes. We also emphasize that future research should examine this issue further, potentially comparing the experiences of abuse in sports between adults and youth.

This study's findings have the potential to inform and improve sexual harassment prevention strategies across the sports sector. By identifying differences in perceptions based on gender and level of sports involvement, the research can guide the development of more targeted and effective educational programs and policies. This is particularly crucial given the ongoing challenges in addressing sexual harassment in sports and the need for evidence-based approaches to create safer environments for all participants. Ultimately, this research not only advances our academic understanding of sexual harassment perceptions in sports but also contributes to the broader societal goal of ensuring that sports remain a positive and empowering experience for all individuals, regardless of their gender or level of participation.

For future recommendations, it is essential to continue raising awareness about sexual harassment in sports and promoting a culture of respect and safety for all athletes, coaches, and staff. Sport organizations should implement comprehensive policies and procedures for addressing sexual harassment, provide training for all members, and establish mechanisms for reporting and investigating incidents. Educational programs should focus on promoting healthy relationships, consent, and bystander intervention. Additionally, ongoing research and evaluation of these initiatives are necessary to ensure their effectiveness in creating safe and inclusive sporting environments.

## Data Availability

The original contributions presented in the study are included in the article/Supplementary Material, further inquiries can be directed to the corresponding author.

## References

[1] Fasting K Chroni S Hervik SE Knorre N. Sexual harassment in sport toward women in three European countries. Int Rev Sociol Sport. (2011) 46(1):76–89. 10.1177/1012690210376295

[2] Parent S Lavoie F Thibodeau MÈ Hébert M Blais M Team PAJ. Sexual violence experienced in the sport context by a representative sample of Quebec adolescents. J Interpers Violence. (2016) 31(16):2666–86. 10.1177/0886260515580366 25873593 PMC5067151

[3] Taylor EA Smith AB Welch NM Hardin R. “You should be flattered!”: woman sport management faculty experiences of sexual harassment and sexism. Women Sport Phys Act J. (2018) 26(1):43–53. 10.1123/wspaj.2017-0038

[4] Taylor EA Hardin R. A gap in the sport management curriculum: an analysis of sexual harassment and sexual assault education in the United States. J Hosp Leis Sport Tour Educ. (2017) 20:65–75. 10.1016/j.jhlste.2017.04.004

[5] Vertommen T Schipper-van Veldhoven NH Hartill MJ Van Den Eede F. Sexual harassment and abuse in sport: the NOC* plNSF helpline. Int Rev Sociol Sport. (2015) 50(7):822–39. 10.1177/1012690213498079

[6] Vertommen T Schipper-van Veldhoven N Wouters K Kampen JK Brackenridge CH Rhind DJ Interpersonal violence against children in sport in The Netherlands and Belgium. Child Abuse Negl. (2016) 51:223–36. 10.1016/j.chiabu.2015.10.006 26516053

[7] Johansson S Lundqvist C. Sexual harassment and abuse in coach–athlete relationships in Sweden. Eur J Sport Soc. (2017) 14(2):117–37. 10.1080/16138171.2017.1318106

[8] Fasting K Brackenridge C. Coaches, sexual harassment and education. Sport Educ Soc. (2009) 14(1):21–35. 10.1080/13573320802614950

[9] Brackenridge C Fasting K. The grooming process in sport: narratives of sexual harassment and abuse. Auto/biography. (2005) 13(1):33–52. 10.1191/0967550705ab016oa

[10] Vertommen T Kampen J Schipper-van Veldhoven N Wouters K Uzieblo K Van Den Eede F. Profiling perpetrators of interpersonal violence against children in sport based on a victim survey. Child Abuse Negl. (2017) 63:172–82. 10.1016/j.chiabu.2016.11.029 27923185

[11] Papp LJ McClelland SI. Too common to count? “mild” sexual assault and aggression among US college women. J Sex Res. (2021) 58(4):488–501. 10.1080/00224499.2020.1778620 32615816

[12] Kim S. Sexual harassment, abuse and intimate relationships between coaches and athletes: a systematic review. Sport Educ Soc. (2024):1–21. 10.1080/13573322.2024.2379941

[13] Lang M Mergaert L Arnaut C Vertommen T. Gender-based violence in sport: prevalence and problems. Eur J Sport Soc. (2023) 20(1):57–78. 10.1080/16138171.2021.2003057

[14] Marks S Mountjoy M Marcus M. Sexual harassment and abuse in sport: the role of the team doctor. Br J Sports Med. (2012) 46(13):905–8. 10.1136/bjsports-2011-090345 22171340

[15] Marshall S McNeil N Seal EL Nicholson M. The “Boys’ club”, sexual harassment, and discriminatory resourcing: an exploration of the barriers faced by women sport officials in Australian basketball. Int Rev Sociol Sport. (2023) 58(6):971–95. 10.1177/10126902221137802

[16] Smith LR Pegoraro A. Media framing of Larry Nassar and the USA gymnastics child sex abuse scandal. J Child Sex Abus. (2020) 29(4):373–92. 10.1080/10538712.2019.1703233 32040384

[17] Fasting K Brackenridge C Walseth K. Consequences of sexual harassment in sport for woman athletes. J Sex Aggress. (2002) 8(2):37–48. 10.1080/13552600208413338

[18] Mountjoy M. ‘Only by speaking out can we create lasting change’: what can we learn from the Dr Larry Nassar tragedy? Br J Sports Med. (2019) 53(1):57–60. 10.1136/bjsports-2018-099403 29936429

[19] Hardin R Taylor EA Sleadd E. Woman students’ experiences of sexual harassment in the sport management internship setting. Sport Manag Educ J. (2021) 15(2):87–94. 10.1123/smej.2020-0021

[20] Chroni S Fasting K. Prevalence of male sexual harassment among woman sports participants in Greece. Inq Sport Phys Educ. (2009) 7(3):288–96.

[21] Fasting K Chroni S Knorre N. The experiences of sexual harassment in sport and education among European woman sports science students. Sport Educ Soc. (2014) 19(2):115–30. 10.1080/13573322.2012.660477

[22] Volkwein KA Schnell FI Sherwood D Livezey A. Sexual harassment in sport: perceptions and experiences of American woman student-athletes. Int Rev Sociol Sport. (1997) 32(3):283–95. 10.1177/1012690297032003005

[23] Tomlinson A Yorganci I. Male coach/woman athlete relations: gender and power relations in competitive sport. J Sport Soc Issues. (1997) 21(2):134–55. 10.1177/019372397021002003

[24] Auweele YV Opdenacker J Vertommen T Boen F Van Niekerk L De Martelaer K Unwanted sexual experiences in sport: perceptions and reported prevalence among Flemish woman student-athletes. Int J Sport Exerc Psychol. (2008) 6(4):354–65. 10.1080/1612197X.2008.9671879

[25] Meyer JW Rowan B. Institutionalized organizations: formal structure as myth and ceremony. Am J Soc. (1977) 83(2):340–63. 10.1086/226550

[26] Nite C Edwards J. From isomorphism to institutional work: advancing institutional theory in sport management research. Sport Manag Rev. (2021) 24(5):815–38. 10.1080/14413523.2021.1896845

[27] Scott WR. Institutions and Organizations. Thousand Oaks, CA: Sage (1995).

[28] Yang Y Konrad AM. Understanding diversity management practices: implications of institutional theory and resource-based theory. Group Organ Manag. (2011) 36(1):6–38. 10.1177/1059601110390997

[29] Wang HK Tseng JF Yen YF. How do institutional norms and trust influence knowledge sharing? An institutional theory. Innovation. (2014) 16(3):374–91. 10.1080/14479338.2014.11081994

[30] Cunningham GB Sagas M. Gender and sex diversity in sport organizations: introduction to a special issue. Sex Roles. (2008) 58(1):3–9. 10.1007/s11199-007-9360-8

[31] Giazitzoglu A. This sporting life: the intersection of hegemonic masculinities, space and emotions among rugby players. Gender Work Organ. (2020) 27(1):67–81. 10.1111/gwao.12367

[32] Davis L Jowett S. Coach–athlete attachment and the quality of the coach–athlete relationship: implications for athlete’s well-being. J Sports Sci. (2014) 32(15):1454–64. 10.1080/02640414.2014.898183 24713087

[33] Gaedicke S Schäfer A Hoffmann B Ohlert J Allroggen M Hartmann-Tews I Sexual violence and the coach–athlete relationship—a scoping review from sport sociological and sport psychological perspectives. Front Sports Act Living. (2021) 3:643707. 10.3389/fspor.2021.643707 34056586 PMC8155665

[34] Sundgot-Borgen J Torstveit MK. Aspects of disordered eating continuum in elite high-intensity sports: disordered eating in elite athletes. Scand J Med Sci Sports. (2010) 20:112–21. 10.1111/j.1600-0838.2010.01190.x 20840569

[35] Anderson ED. The maintenance of masculinity among the stakeholders of sport. Sport Manag Rev. (2009) 12(1):3–14. 10.1016/j.smr.2008.09.003

[36] Fejgin N Hanegby R. Gender and cultural bias in perceptions of sexual harassment in sport. Int Rev Sociol Sport. (2001) 36(4):459–78. 10.1177/101269001036004006

[37] Owton H Sparkes AC. Sexual abuse and the grooming process in sport: learning from Bella’s story. Sport Educ Soc. (2017) 22(6):732–43. 10.1080/13573322.2015.1063484

[38] Bisgaard K Støckel JT. Athlete narratives of sexual harassment and abuse in the field of sport. J Clin Sport Psychol. (2019) 13(2):226–42. 10.1123/jcsp.2018-0036

[39] Bartholomew KJ Ntoumanis N Ryan RM Thøgersen-Ntoumani C. Psychological need thwarting in the sport context: assessing the darker side of athletic experience. J Sport Exerc Psychol. (2011) 33(1):75–102. 10.1123/jsep.33.1.75 21451172

[40] Reel JJ Crouch E. # MeToo: uncovering sexual harassment and assault in sport. J Clin Sport Psychol. (2019) 13(2):177–9. 10.1123/jcsp.2018-0078

[41] Tam A Kerr G Stirling A. Influence of the# MeToo movement on coaches’ practices and relations with athletes. Int Sport Coach J. (2020) 8(1):1–12. 10.1123/iscj.2019-0081

[42] Burke AM. Raising the bar: increasing protection for athletes in the Olympic movement from sexual harassment and abuse. J Legal Asp Sport. (2021) 31:60. 10.18060/24920

[43] Guy S Zach S. The person who loves me the most is also the person who hurts me the most: power relations between a single coach and an athlete from the athletes’ point of view. Spirit Sport (Ruach Ha’Sport). (2023) 9:83–110. (Hebrew).

[44] Sand TS Fasting K Chroni S Knorre N. Coaching behavior: any consequences for the prevalence of sexual harassment? Int J Sports Sci Coach. (2011) 6(2):229–41. 10.1260/1747-9541.6.2.229

[45] Zach S Guy S Ben-Yeheskel R Grosman-Rimon L. Clear yet crossed: athletes’ retrospective reports of coach violence. Behav Sci. (2024) 14(6):486. 10.3390/bs14060486 38920818 PMC11200403

[46] Fejgin N Hanegby R. Is it just sport? Perceptions and experiences of sexual harassment in sport. Dapim. (2000) 30:106–29. (Hebrew).

[47] Brackenridge C. Spoilsports: Understanding and Preventing Sexual Exploitation in Sport. Routledge (2001). 10.4324/9780203478936

[48] Mohipp C Senn CY. Graduate students’ perceptions of contrapower sexual harassment. J Interpers Violence. (2008) 23(9):1258–76. 10.1177/0886260508314299 18314508

[49] Hassall CE Bringer JD Johnston LH Brackenridge CH. Coach and athlete perceptions of ambiguous behaviors and sexual harassment. J Sport Pedagog. (2002) 2:1–21.

[50] Carstensen G. Sexual harassment reconsidered: the forgotten grey zone. NORA Nord J Fem Gend Res. (2016) 24(4):267–80. 10.1080/08038740.2017.1292314

[51] Ahmed MD Cardinal BJ Khan S Khan BA Begum S. Male coaches’ sexual harassment, abuse, and assault as perceived by woman athletes in India and Pakistan. Phys Educ. (2022) 79(6):629–46. 10.18666/TPE-2022-V79-I6-10984

[52] Johansson S. Coach–athlete Sexual Relationships: If no Means no, Does yes Mean yes? Moral Panic in Physical Education and Coaching. Stockholm: Routledge (2017). p. 104–19.

[53] Quick JC McFadyen M. Sexual harassment: have we made any progress? J Occup Health Psychol. (2017) 22(3):286. 10.1037/ocp0000054 27732009

[54] Wood L Hoefer S Kammer-Kerwick M Parra-Cardona JR Busch-Armendariz N. Sexual harassment at institutions of higher education: prevalence, risk, and extent. J Interpers Violence. (2021) 36(9-10):4520–44. 10.1177/0886260518791228 30071790 PMC10676016

[55] Saul J. Stop thinking so much about ’sexual harassment’. J Appl Philos. (2014) 31(3):307–21. 10.1111/japp.12058

